# Development and validation of an individualized nomogram for predicting distant metastases in gastric cancer using a CT radiomics-clinical model

**DOI:** 10.3389/fonc.2024.1476340

**Published:** 2024-11-29

**Authors:** Hui-Bin Xue, Mei-Li Liang, Huang-Zhen Xu, Chen-Yu Wang, Tian-Wen Xu, Ai-Yue Zhao

**Affiliations:** ^1^ Department of Digestive Tumor, The Second Affiliated Hospital of Fujian Medical University, Quanzhou, China; ^2^ Department of Gynecology and Obstetrics, The Second Affiliated Hospital of Fujian Medical University, Quanzhou, China; ^3^ Department of Oncology, The Second Affiliated Hospital of Fujian Medical University, Quanzhou, China

**Keywords:** gastric cancer, distant metastases, radiomics, nomogram, computed tomography

## Abstract

**Purpose:**

This study aimed to develop and validate a model for accurately assessing the risk of distant metastases in patients with gastric cancer (GC).

**Methods:**

A total of 301 patients (training cohort, n = 210; testing cohort, n = 91) with GC were retrospectively collected. Relevant clinical predictors were determined through the application of univariate and multivariate logistic regression analyses. Then the clinical model was established. Venous phase computed tomography (VPCT) images were utilized to extract radiomic features, and relevant features were selected using univariate analysis, Spearman correlation coefficient, and the least absolute shrinkage and selection operator (Lasso) regression. Subsequently, radiomics scores were calculated based on the selected features. Radiomics models were constructed using five machine learning algorithms according to the screened features. Furthermore, separate joint models incorporating radiomic features and clinically independent predictors were established using traditional logistic regression algorithms and machine learning algorithms, respectively. All models were comprehensively assessed through discrimination, calibration, reclassification, and clinical benefit analysis.

**Results:**

The multivariate logistic regression analysis revealed that age, histological grade, and N stage were independent predictors of distant metastases. The radiomics score was derived from 15 selected features out of a total of 944 radiomic features. The predictive performance of the joint model 1 [AUC (95% CI) 0.880 (0.811-0.949)] constructed using logistic regression is superior to that of the joint model 2 [AUC (95% CI) 0.834 (0.736-0.931)] constructed using SVM algorithm. The joint model 1 [AUC(95% CI) 0.880(0.811-0.949)], demonstrated superior performance compared to the clinical model [AUC(95% CI) 0.781(0.689-0.873)] and radiomics model [AUC(95% CI) 0.740(0.626-0.855), using LR algorithm]. The NRI and IDI values for the joint model 1 and clinical model were 0.115 (95% CI 0.014 -0.216) and 0.132 (95% CI 0.093-0.171), respectively; whereas for the joint model 1 and LR model, they were found to be 0.130 (95% CI 0.018-0.243) and 0.116 (95% CI 0.072-0.160), respectively. Decision curve analysis indicated that the joint model 1 exhibited a higher clinical net benefit than other models.

**Conclusions:**

The nomogram of the joint model, integrating radiomic features and clinically independent predictors, exhibits robust predictive capability for early identification of high-risk patients with a propensity for distant metastases of GC.

## 1 Introduction

Gastric cancer (GC) is a prevalent malignancy globally, ranking fifth in terms of its incidence and fourth in terms of its mortality rate (1). It is well-established that the prognosis of GC varies significantly depending on the treatment approach employed at different stages (2). Studies have reported that at initial diagnosis, distant metastases are observed in approximately 21.9-44.6% of patients with GC (3, 4), leading to an unfavorable prognosis with a median overall survival of merely 5 months and a 5-year survival rate as low as 3.9% (5). Therefore, early identification and effective intervention targeting high-risk patients with distant metastases can substantially enhance patient outcomes.

The primary modality for identifying distant metastases in clinical practice is computed tomography (CT) examination, which offers advantages such as high spatial resolution, strong non-invasiveness, and robust technical support for image processing. However, the CT diagnosis of distant metastasis in GC exhibits characteristics of high specificity but low sensitivity (6). MRI and PET-CT serve as adjunctive modalities for detecting distant metastasis in conjunction with CT, yet their sensitivity remains limited (6). Moreover, existing imaging techniques can only identify present distant metastases and cannot predict the risk of future occurrences. Therefore, clinicians encounter considerable difficulty in precisely identifying distant metastases, highlighting the pressing requirement for the development of an innovative and accurate predictive approach that can serve as a supplementary diagnostic tool in detecting distant metastases among recently diagnosed GC patients.

Radiomics, an increasingly prominent field in recent years, employs high-throughput extraction of quantitative features to convert medical images into high-dimensional and minable data, followed by comprehensive data analysis for decision support (7). By comprehensively extracting quantitative features from regions of interest, radiomics can discern subtle differences in medical images that may elude human perception and quantify the extent of heterogeneity observed in neoplasms (8). Moreover, the quantitative features obtained from images can provide insights into biological aspects including cellular morphology, gene expression, and molecular properties (9). These features are relatively independent yet interconnected with traditional clinical and molecular attributes, thereby enhancing the accuracy of evidence-based medicine (10, 11). Prior research has showcased the resilience of radiomics models that employ CT scans to effectively forecast lymph node metastasis, assess ovarian metastasis, and detect peritoneal and omental metastasis among patients with GC (12–15). These findings offer promising prospects for utilizing radiomic features and clinical data to prognosticate the risk of distant metastases in individuals with GC.

Hence, we hypothesize that radiomics could be a valuable asset in forecasting the risk of distant metastases in GC. The primary aim of this study was to establish a predictive model for distant metastases using CT-based radiomic features and clinical data, with the ultimate goal of developing an individualized nomogram to offer an influential instrument for personalized therapy of patients with GC.

## 2 Materials and methods

### 2.1 Patients

The present study retrospectively screened all cases of GC initially diagnosed at the Second Affiliated Hospital of Fujian Medical University from January 2018 to December 2022, as retrieved from the database.

The stated criteria were utilized for the purpose of inclusion: (1) histologically confirmed primary GC; (2) comprehensive evaluation of distant metastases using whole-body 18F-FDG-PET-CT or contrast-enhanced CT scans of the chest, abdomen, and pelvis, MRI of the brain, and radionuclide bone imaging prior to treatment initiation. Exclusion criteria included: (1) coexistence with other primary malignant tumors; (2) Siewert type I esophagogastric junction tumors; (3) insufficient clinical data; and (4) obvious artifacts or poor gastric distension on CT images. A total of 301 patients meeting these criteria were randomly allocated to either a training cohort or a testing cohort, with a distribution ratio of 7:3.

**Figure 1:**
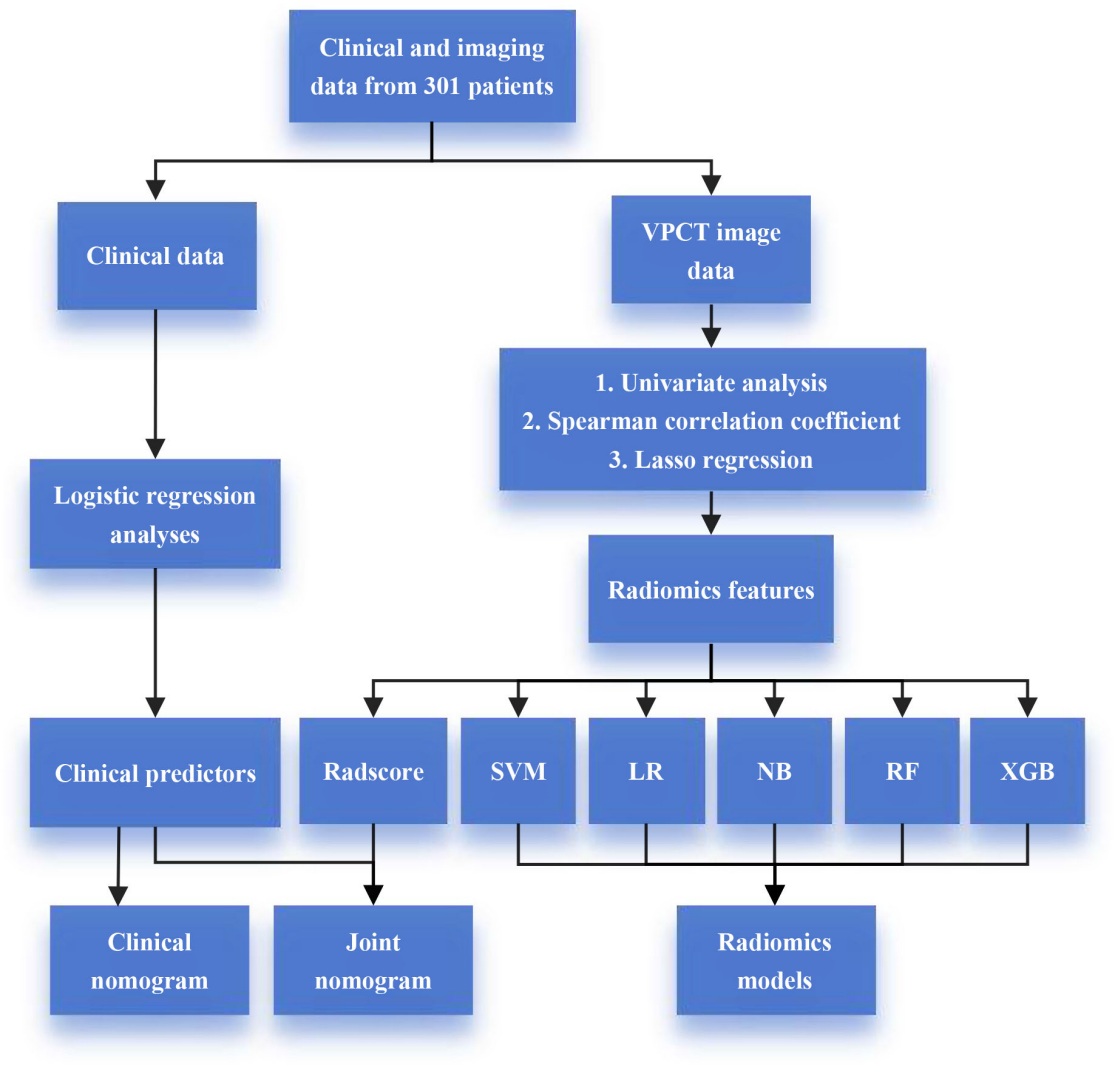
Flowchart of the study.

This study received ethical approval from the Ethics Committee of the Second Affiliated Hospital of Fujian Medical University, and the need for obtaining informed consent was waived. The research flowchart is illustrated in **Figure 1**.

### 2.2 Data and images collection

The collected data encompassed clinical characteristics and VPCT images. Clinical characteristics of patients were extracted from the medical record system, including age, gender, tumor location, tumor size, histological classification and grade, T stage, N stage, M stage, CEA and CA19-9 levels, neutrophil-to-lymphocyte ratio (NLR), platelet-to-lymphocyte ratio (PLR), as well as metastatic site information. Distant metastasis is defined as non-regional lymph node metastasis or distant organ metastasis confirmed by biopsy. For gastric cancer patients initially diagnosed with distant metastasis via computed tomography (CT), a subset may forgo biopsy. However, the presence of metastatic disease can be confirmed if there is a change in size of the lesions during post-treatment follow-up, leading to classification as stage M1.

The CT images are acquired from the radiology department and saved in the Digital Imaging and Communication in Medicine (DICOM) format. All subjects underwent comprehensive multi-phase contrast-enhanced CT examinations, including arterial, venous, and delayed phases, in addition to non-enhanced CT scans, prior to their respective treatments. The CT examination was performed using a 128-slice Philips Brilliance iCT and a Siemens dual-source CT scanner. Other scanning parameters involved setting the tube voltage at 120 kV, adjusting the tube current within the range of 180 to 540 mA, maintaining a slice thickness of 2 mm, utilizing an image matrix size measuring 512×512, and determining pixel spacing as 0.765625×0.765625.

### 2.3 Image segmentation and radiomic features extraction

Image segmentation was performed using ITK-SNAP software (version 3.6.0; http://www.itksnap.org). A medical oncologist with 5 years of expertise in oncology manually segmented the regions of interest (ROI) for all subjects’ lesions, which were subsequently reviewed by a radiologist possessing a decade of professional expertise. Both doctors were blinded to the patients’ clinical conditions. The segmented CT image files were saved in Neuroimaging Informatics Technology Initiative(NIFTI) format. When conducting feature extraction, the ROI images underwent normalization and resampling to achieve a pixel spacing of 2.0mm×2.0mm×2.0mm, thereby ensuring accurate pixel size and slice thickness. Pyradiomics (16), an open-source Python package accessible at the following link (https://pyradiomics.readthedocs.io/en/latest/), was used for feature extraction.

Feature extraction was facilitated by employing Laplacian of Gaussian (LoG) and wavelet filters, which are renowned for their efficacy in enhancing relevant information and reducing noise within digital image processing. The ROIs can yield five distinct types of radiomic features: (1) first-order statistical features, (2) shape and size features, (3) texture features, (4) LoG features, and finally, (5) wavelet features.

To guarantee the reproducibility and reliability of radiomics features, 30 cases were randomly selected. One month after the initial segmentation, the same oncologist (Observer 1) and the same radiologist (Observer 2) resegmented the ROI to assess the intra- and inter-observer reproducibility of the extracted features. Only features with an intra-class correlation coefficient (ICC) greater than 0.75 for both inter- and intra-observer agreements were considered stable and selected for further analysis.

### 2.4 Feature selection and radiomics score construction

**Figure 2:**
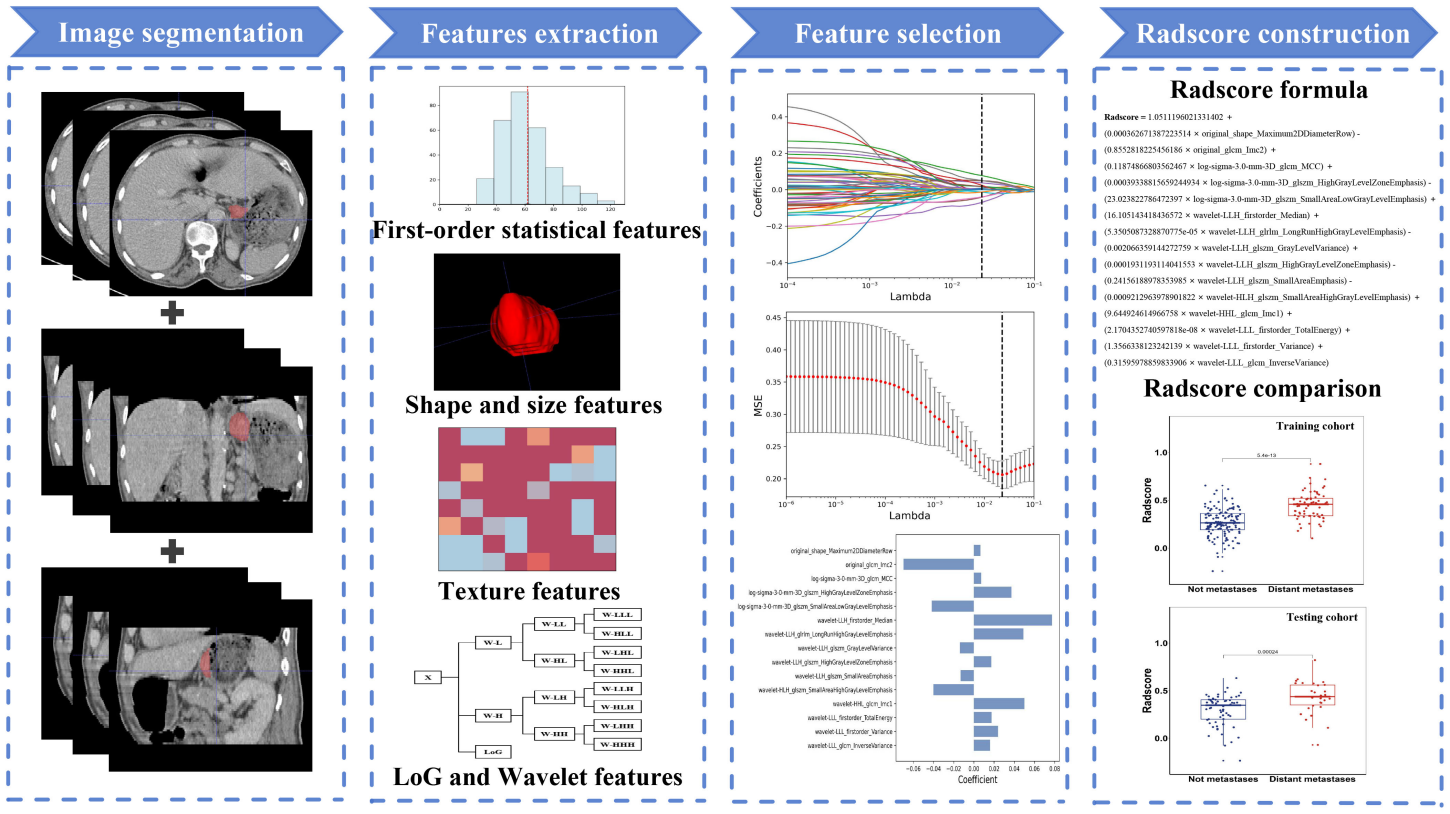
The methodology for creating the radiomics score.

In the feature selection process, we employed univariate analysis to eliminate irrelevant features. The Spearman correlation coefficient was utilized to identify feature pairs exhibiting a high degree of correlation. Feature pairs characterized by a correlation coefficient absolute value exceeding 0.9 were deemed to have a strong correlation and thus, only one of the two feature was retained. The remaining features were standardized using the formula (X − μ)/σ, where μ is the mean and σ is the standard deviation of each feature. Subsequently, the least absolute shrinkage and selection operator regression (Lasso) was applied to identify radiomic features that exhibit the strongest correlation with distant metastasis in GC. The model’s correlation coefficients and constants were calculated, leading to derivation of the radiomics scoring formula. **Figure 2** illustrates the methodology for creating the radiomics score (radscore).

### 2.5 Clinical model construction

We employed univariate and multivariate logistic regression analyses in order to ascertain the clinically independent predictors. Subsequently, we constructed clinical nomograms incorporating these independent predictors.

### 2.6 Radiomics models construction

We utilize selected radiomic features to construct radiomics models employing various machine learning algorithms, including support vector machines (SVM), logistic regression (LR), random forests (RF), naive Bayes (NB), and extreme gradient boosting models (XGB). We then compare the effectiveness of these models to determine the optimal algorithm.

### 2.7 Radiomics-clinical model construction

Relevant clinical predictors that showed statistical significance in the univariate regression analysis were incorporated into a logistic multivariate regression analysis along with radscore. Afterwards, we developed the joint model 1 by including variables with a P-value below 0.05. Furthermore, we incorporated both clinical features and radiomic features into the feature selection process, identifying the clinical and radiomic features most strongly associated with distant metastasis of GC through feature selection. Subsequently, we constructed joint models using the aforementioned five machine learning algorithms and determined the optimal machine learning joint model 2 by comparing the effectiveness of these models. Finally, by comparing the predictive performance of joint model 1 and joint model 2, we identified the best joint model and compared it with the clinical model and the best radiomics model.

### 2.8 Model evaluation

The effectiveness of the models was gauged through the AUC, indicating the area under the receiver operating characteristic (ROC) curve. To compare the AUC values across different models, we employed the DeLong test. The calibration of the best joint model was evaluated using a calibration curve and Hosmer-Lemeshow test. Net reclassification improvement (NRI) and integrated discrimination improvement (IDI) were computed to compare performance across various models. Additionally, decision curve analysis (DCA) was conducted to evaluate the practical value of our models by quantifying net benefit at various threshold probabilities. Ultimately, the sensitivity and specificity of the best joint model were determined and compared with those of conventional computed tomography (CT) for diagnostic accuracy.

### 2.9 Statistical analysis

The statistical analysis was conducted using IBM SPSS software (version 27.0), Python software (version 3.11.4), and R software (version 4.4.0; https://www.r-project.org). Clinical baseline characteristics were compared between the training cohort and testing cohort, employing the Chi-square test for categorical variables and the Mann-Whitney test for continuous variables. Additionally, the Mann-Whitney test was utilized to assess radscore consistency between the training and testing cohort. A statistical significance was determined when the p-value was less than 0.05 on both sides.

## 3 Results

### 3.1 Clinical characteristics

Among the 301 eligible cases, 202 presented no distant metastasis while 99 exhibited distant metastasis at initial diagnosis, resulting in an incidence rate of 32.9%. Peritoneal dissemination emerged as the predominant location for distant metastasis (42.4%), closely trailed by hepatic involvement (39.4%) and non-regional lymph node infiltration (30.3%). Less frequent sites of metastasis included the lung (14.1%), adrenal glands (6.1%), bone (5.1%), pancreas (5.1%), spleen (2.0%), abdominal wall (2.0%), ovary (2.0%), and brain (1.0%). The distribution of metastases was characterized by 57 cases (57.6%) with involvement of a single organ and 43 cases (43.4%) with involvement of multiple organs, with non-regional lymph nodes being counted as one organ.

The entire cohort was divided into two cohorts, namely a training cohort (n=210) and a testing cohort (n=91), using a random allocation method with a ratio of 7:3. In the training cohort, distant metastasis was identified in only 69 cases (32.9%), whereas 141 cases (67.1%), exhibited no indications of distant metastasis. Similarly, within the testing cohort, there were 30 cases (33.0%) with distant metastasis and 61 cases (67.0%) without distant metastasis observed. Comparison of clinical characteristics between these two cohorts revealed no significant differences in baseline characteristics (**Table 1**), indicating satisfactory data division outcomes.

**Table 1:** Comparison of baseline characteristics between the training and testing cohorts in patients with gastric cancer.

Characteristics	Training cohort(n = 210)	Testing cohort(n = 91)	P-value
**Length (cm)**	4.561 ± 2.168	4.352 ± 1.982	0.453
**NLR**	4.292 ± 2.103	4.193 ± 1.747	0.877
**PLR**	181.063 ± 58.675	183.521 ± 58.684	0.759
**Age (years)**			0.305
≥75	32	18	
60-74	110	37	
45-59	60	31	
≤44	8	5	
**Gender**			0.164
Female	60	19	
Male	150	72	
**Location**			0.730
Cardia - fundus	77	28	
Body	45	23	
Antrum	69	30	
2/3-whole stomach	19	10	
**Histology**			0.418
adenocarcinoma	172	78	
others	38	13	
**Grade**			0.843
G_1_/G_2_	93	37	
G_3_	100	46	
G_x_	17	8	
**Stage T**			0.321
T_1_/T_2_	24	7	
T_3_	109	43	
T_4_	77	41	
**Stage N**			0.633
N_0_	39	12	
N_1_	31	17	
N_2_	68	30	
N_3_	72	32	
**CEA**			0.472
Normal	146	67	
Elevated	64	24	
**CA19-9**			0.718
Normal	148	66	
Elevated	62	25	

NLR, neutrophil-to-lymphocyte ratio; PLR, platelet-to-lymphocyte ratio; CEA, carcinoembryonic antigen; CA19-9, carbohydrate antigen 19-9.

### 3.2 Features selection and radiomics score

A total of 944 radiomic features were extracted from the ROIs, and after conducting ICC, univariate analysis, Spearman correlation coefficient, and applying the Lasso regression algorithm, we ultimately selected 15 features that exhibited the strongest association with distant metastasis of GC. The calculation formula for radscore is illustrated in **Figure 2**.

In the training cohort, there was a significant difference in radscores between patients with and without distant metastasis (mean ± SD: 0.445 ± 0.141 vs 0.272 ± 0.145; z = -7.216, P < 0.001). A similar trend was observed in the testing cohort where patients with distant metastasis had higher radscores compared to those without (mean ± SD: 0.429 ± 0.173 vs 0.294 ± 0.163; z = -3.672, P < 0.001). Furthermore, the radscore of the training cohort was 0.329 ± 0.165, while the radscore of the testing cohort was 0.338 ± 0.177 (z = -1.001, P = 0.317), indicating satisfactory data division outcomes.

### 3.3 Development and validation of the clinical model

The univariate logistic regression analysis revealed significant differences in age, histological grade, T stage, N stage, CEA, and CA19-9 (**Table 2**). Subsequently, a multivariate logistic regression analysis was conducted on the aforementioned independent predictors. The results demonstrated that age, histological grade, and N stage were clinically independent predictors (P<0.05).

**Table 2:** Univariate and multivariate logistic regression analysis.

Characteristics	Univariate regression		Multivariate regression	
	OR (95%CI)	P-value	OR (95%CI)	P-value
Age				
≥75	Reference			
60-74	0.803 [0.408, 1.615]	0.530	0.858 [0.362, 2.064]	0.730
45-59	0.955 [0.462, 2.004]	0.900	1.408 [0.567, 3.596]	0.470
≤44	4.368 [1.233, 18.099]	**0.030**	5.168 [1.114, 26.844]	**0.040**
Gender				
Female	Reference			
Male	0.999 [0.582, 1.742]	1.000		
Location				
Cardia-fundus	Reference			
Body	1.253 [0.641, 2.433]	0.510		
Antrum	1.633 [0.908, 2.959]	0.100		
2/3-whole stomach	1.379 [0.558, 3.273]	0.470		
Length	1.020 [0.909, 1.141]	0.730		
Histology				
adenocarcinoma	Reference			
others	0.920 [0.471, 1.733]	0.800		
Grade				
G_1_/G_2_	Reference		Reference	
G_3_	7.418 [4.136, 13.936]	**<0.001**	4.554 [2.314, 9.330]	**<0.001**
G_x_	1.662 [0.502, 4.768]	0.370	0.698 [0.179, 2.384]	0.580
Stage T				
T_1_/T_2_	Reference		Reference	
T_3_	4.050 [1.347, 17.528]	**0.030**	0.830 [0.199, 4.440]	0.810
T_4_	6.863 [2.268, 29.819]	**<0.001**	0.869 [0.197, 4.829]	0.860
Stage N				
N_0_	Reference		Reference	
N_1_	5.333 [1.559, 24.681]	**0.010**	2.747 [0.614, 15.450]	0.210
N_2_	9.290 [3.109, 40.131]	**<0.001**	3.794 [1.021, 18.986]	0.070
N_3_	13.714 [4.641, 58.920]	**<0.001**	5.723 [1.541, 28.790]	**0.020**
CEA				
Normal	Reference		Reference	
Elevated	1.894 [1.129, 3.174]	**0.020**	1.539 [0.734, 3.230]	0.250
CA19-9				
Normal	Reference		Reference	
Elevated	2.397 [1.429, 4.032]	**<0.001**	1.358 [0.651, 2.824]	0.410
NLR	1.047 [0.928, 1.182]	0.450		
PLR	0.999 [0.995, 1.003]	0.590		
Radscore (per 0.1 increase)	2.177 [1.769, 2.741]	<0.001	2.069 [1.622, 2.715]	<0.001

OR, odds ratio; CI, confidence interval; NLR, neutrophil-to-lymphocyte ratio; PLR, platelet-to-lymphocyte ratio; CEA, carcinoembryonic antigen; CA19-9, carbohydrate antigen 19-9.

Bold values: p < 0.05.

**Figure 3:**
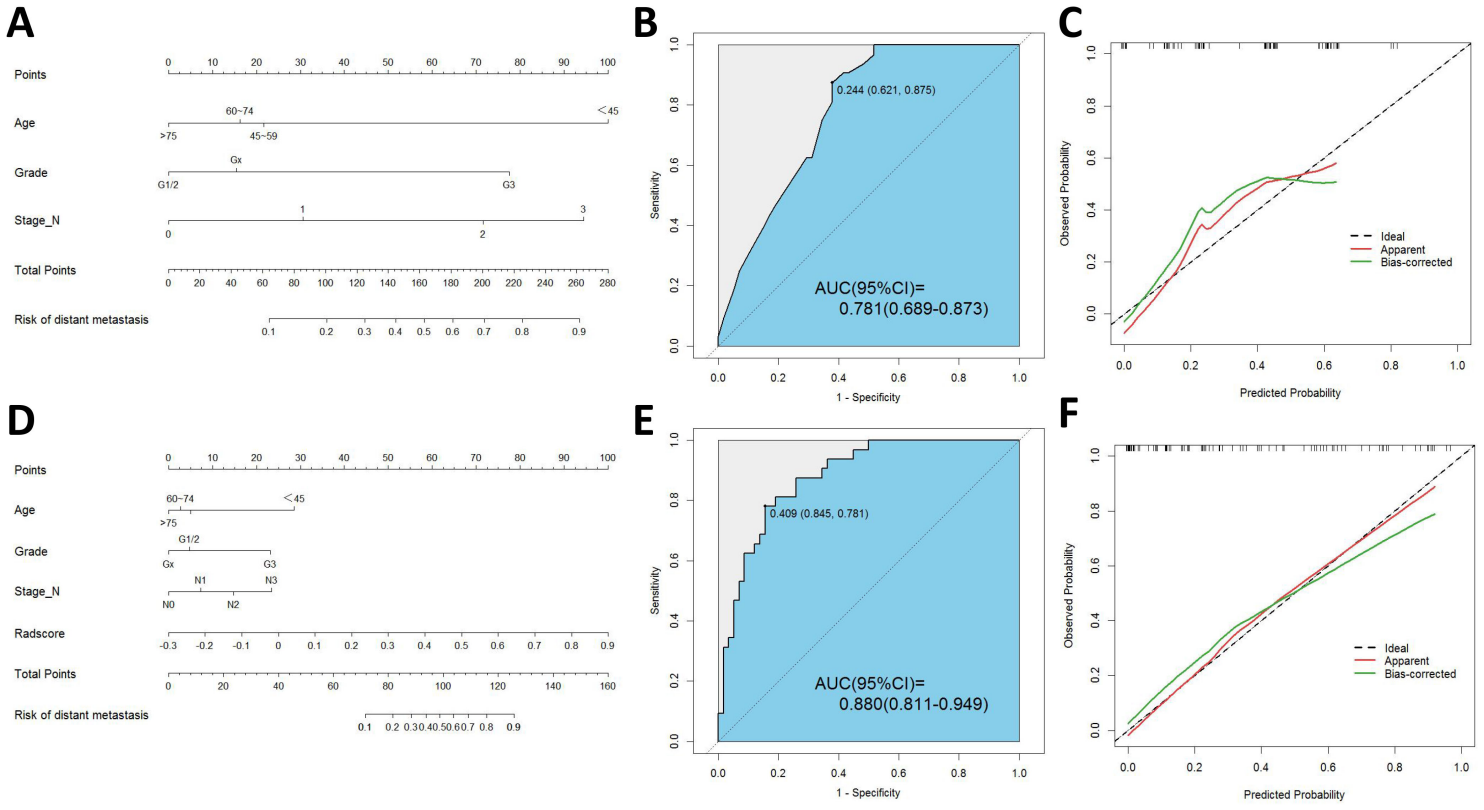
**(A)** Nomogram of clinical model. **(B)** ROC curve of clinical model. **(C)** Calibration curve of clinical model. **(D)** Nomogram of radiomics-clinical model. **(E)** ROC curve of radiomics-clinical model. **(F)** Calibration curve of radiomics-clinical model.

Based on these predictors obtained from the final screening, we developed a clinical prediction model. The AUC of the training cohort was found to be 0.797 (95% CI 0.738-0.856). Furthermore, the AUC of the testing cohort was found to be 0.781 (95% CI 0.689-0.873). **Figure 3** presents the nomogram along with ROC and calibration curves illustrating our clinical prediction model.

### 3.4 Development and validation of the radiomics models

**Figure 4:**
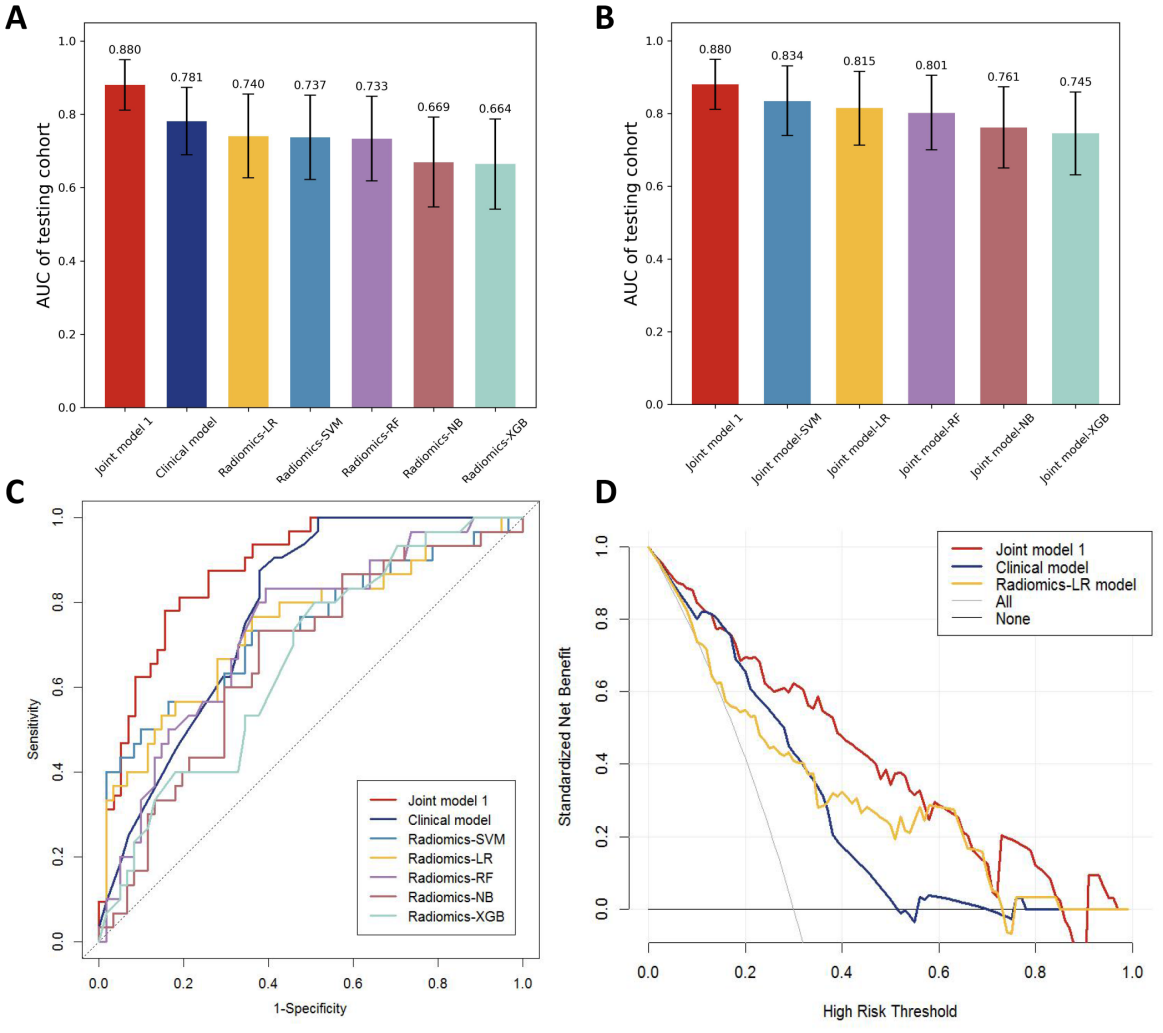
**(A)** AUC comparison of testing cohorts for all models. **(B)** AUC comparison of testing cohorts for all joint models. **(C)** ROC comparison of testing cohorts for all models. **(D)** DCA curve.

Different machine learning algorithms, including support vector machine (SVM), logistic regression (LR), Random forest (RF), Naive Bayes (NB), and extreme gradient enhancement model (XGB), were employed to develop the radiomics models. **Figure 4A** presents the AUC results of all models in the testing cohort except for the machine learning joint models, revealing that the LR model exhibits the best performance among the radiomics models.

### 3.5 Development and validation of the radiomics–clinical model

The univariate logistic regression analysis revealed a statistically significant difference in radscore between patients with distant metastasis and those without (P < 0.001). The logistic multivariate regression analysis incorporated the radscore and clinical characteristics that demonstrated statistical significance in the univariate regression analysis. The results revealed significant statistical associations (P < 0.05) between age, histological grade, N stage, and radscore (**Table 2**), leading to the establishment of the joint model 1. In the training cohort, the AUC was 0.865 (95% CI 0.812-0.918); while in the testing cohort, the AUC was 0.880(95% CI 0.811-0.949).

By incorporating both clinical and radiomic features into the feature selection process, we identified 22 features with the strongest association with distant metastasis of GC after the selection. The clinical features included age, histological grade, N stage, and CEA, while the radiomic features included original_glcm_Imc2, log-sigma-3-0-mm-3D_firstorder_Median, log-sigma-3-0-mm-3D_glcm_MCC, log-sigma-3-0-mm-3D_glrlm_ShortRunLowGrayLevelEmphasis, log-sigma-3-0-mm-3D_glszm_HighGrayLevelZoneEmphasis, log-sigma-3-0-mm-3D_glszm_SmallAreaLowGrayLevelEmphasis, wavelet-LLH_firstorder_Median, wavelet-LLH_glrlm_LongRunHighGrayLevelEmphasis, wavelet-LLH_glszm_GrayLevelVariance, wavelet-LLH_glszm_HighGrayLevelZoneEmphasis, wavelet-LLH_glszm_SmallAreaEmphasis, wavelet-LHL_firstorder_Median, wavelet-LHL_glszm_ZoneVariance, wavelet-HLH_glszm_SmallAreaHighGrayLevelEmphasis, wavelet-HHL_glcm_Imc1, wavelet-LLL_firstorder_TotalEnergy, wavelet-LLL_firstorder_Variance, and wavelet-LLL_glcm_InverseVariance. Using the aforementioned features, we constructed five machine learning joint models. **Figure 4B** presents the AUC of joint model 1 and all machine learning joint models in the testing cohort, demonstrating that the SVM algorithm-built joint model 2 performed the best. In the training cohort, the AUC was 0.842 (95% CI 0.780-0.904); in the testing cohort, the AUC was 0.834 (95% CI 0.736-0.931). The DeLong test indicated that, compared to joint model 2, joint model 1 had superior discriminative performance (P < 0.05). **Figure 3** presents the nomogram along with ROC and calibration curves illustrating our radiomics-clinical prediction model 1.

### 3.6 Evaluation of models

The performance of the clinical model, radiomics models, and joint model 1 was compared in **Figure 4C**. The DeLong test demonstrated that the joint model 1 exhibited significantly better discriminatory capability in comparison to both the clinical model and radiomics models (P < 0.05). The calibration curve indicated a good agreement between predicted and observed values for the joint model 1. Furthermore, the Hosmer-Lemeshow test did not find any statistically significant difference for the joint model 1 (p = 0.5419), suggesting a well-fitted joint model. The NRI and IDI values for the joint model 1 and clinical model were 0.115 (95% CI 0.014 -0.216) and 0.132 (95% CI 0.093-0.171), respectively; whereas for the joint model 1 and LR model, they were found to be 0.130 (95% CI 0.018-0.243) and 0.116 (95% CI 0.072-0.160), respectively. These results indicate a significant improvement in predictive power when comparing the joint model 1 to both the clinical model and LR model. The decision curve analysis depicted in **Figure 4D** demonstrates that across various threshold probabilities, net benefit is better for the joint model 1 than for other models. The sensitivity and specificity of CT for the diagnosis of distant metastasis are detailed in **Table 3**. The sensitivity of CT is 65.7%, with a specificity of 98.5%. In contrast, the sensitivity of the joint model 1 reaches 78.1%, and its specificity is 84.5%.

**Table 3:** The sensitivity and specificity of M staging by CT using histological examination as the reference standard.

Histological staging	N	CT staging		Sensitivity (%)	Specificity (%)
		M0	M1		
**M0**	202	199	3	65.7	98.5
**M1**	99	34	65		
**Total**	301	233	68		

M0: Patients without distant metastasis; M1: Patients with distant metastasis.

## 4 Discussion

Through integration of clinical data and radiomic features, we have successfully developed and validated a nomogram model to precisely forecast the occurrence of distant metastases in gastric cancer. This constructed nomogram can serve as a user-friendly and non-invasive tool for individualized treatment of GC patients.

To construct the most precise prediction model, we developed a diverse range of models including clinical model, radiomics models, and joint model. The clinical model exhibited strong performance (AUC = 0.781). Out of the different machine learning models assessed, the LR model demonstrated exceptional performance with an AUC score of 0.740, slightly lower than that of the clinical model; however, statistical analysis using Delong test revealed no significant difference in AUC between these two models (p > 0.05). Among the joint models 2 constructed using machine learning algorithms, the SVM algorithm demonstrated the best performance. However, compared to joint model 2, joint model 1 achieved superior performance (AUC = 0.880, Delong test, p < 0.05). Furthermore, compared to radiomic models built using machine learning algorithms, the predictive performance of the machine learning joint models was enhanced. These results indicate that integrating radiomics with clinical features enhances diagnostic efficiency within this prediction model framework. Furthermore, we employed DCA curves along with NRI and IDI metrics to comprehensively evaluate various models’ performances. While ROC curves evaluate predictive accuracy solely based on discrimination ability, DCA curves offer valuable insights into the potential drawbacks and advantages associated with false negatives and false positives (17). NRI quantifies differences in correct classification rates between two classifiers by measuring variations in sensitivity and specificity sums (18). Similarly to NRI, IDI measures gaps in prediction probabilities (19). Both indicators are suitable for comparative analyses among different models. In clinical practice, the principal modality for detecting distant metastasis in gastric cancer patients is CT examination. However, studies by Leeman, Feng, Pan, et al. (20–22), have reported that the overall sensitivity of CT in diagnosing distant metastasis varies from 14.3 to 59.1%, with a specificity ranging from 89.6 to 99.8%. In the present study, the sensitivity and specificity of CT for diagnosing distant metastasis were 65.7% and 98.5%, respectively, whereas our joint model exhibited a sensitivity of 78.1% and a specificity of 84.5%. The sensitivity of our model in diagnosing distant metastasis is significantly higher than that of CT scans, which implies a substantial reduction in the rate of missed diagnoses in clinical practice. The specificity of our model is only slightly lower than that of CT scans, suggesting its potential as an auxiliary diagnostic tool to complement CT scans and reduce the rate of false positives.

Radiomics has found extensive application in the investigation of malignancy, including lung cancer, colorectal cancer, thyroid cancer, and gastric cancer (23–26). Moreover, at the microscopic scale, radiomics is employed to evaluate the immune environment and immunotherapy response of tumors (27). In previous studies on gastric cancer, radiomics has been applied to predict lymph node metastasis, peritoneal metastasis, response to neoadjuvant chemotherapy, and long-term survival (12, 14, 28, 29). Gao developed a CT-based radiomic model for predicting lymph node metastasis in gastric cancer with excellent discriminative ability (AUC = 0.89) (12). Dong successfully employed CT radiomics to accurately identify occult peritoneal metastases in advanced gastric cancer patients both in the training set (AUC = 0.958) and test set (AUC = 0.941) (14). Radiomics has also been employed to forecast neoadjuvant chemotherapy response in locally advanced gastric cancer (AUC = 0.736) (28). These results emphasize the considerable potential of radiomics in the development of predictive models. Similarly, our study shows that the joint model achieves excellent accuracy in predicting distant metastases in GC patients, with an AUC of 0.865 for the training cohort and 0.880 for the testing cohort.

Utilizing multivariate logistic regression analysis, we identified age, histological grade, and N stage as independent clinical predictors for distant metastasis in GC patients. Earlier research has documented an increased prevalence of lymph node metastasis among gastric cancer patients in the younger age group (30, 31), suggesting that increased lymph node metastasis in younger patients may contribute to distant metastasis. Furthermore, our findings indicate that patients with poorly differentiated gastric cancer are more prone to developing distant metastases compared to those with moderately and highly differentiated tumors, potentially due to the enhanced growth capacity and invasive potential of poorly differentiated gastric cancer tissues in infiltrating surrounding tissues, capillaries, and lymphatics. Our study incorporated systemic immunoinflammatory markers, including NLR and PLR, as clinically independent predictors for distant metastases. This is due to the strong correlation between tumor development and advancement and inflammation, wherein inflammatory cells facilitate cancer cell proliferation, angiogenesis, and invasion (32). Neutrophils modify the tumor microenvironment and secrete inflammatory mediators to promote tumor cell proliferation, invasion, and metastasis (33). Platelet activation acts as a chemical attractant inducing cancer cell metastasis (34). Lymphocytes have a significant impact on suppressing the growth and spread of cancer cells by employing cytokine-induced cytotoxicity, thereby contributing to effective immune responses against malignancies (35). According to Osama et al., NLR and PLR are linked to gastric cancer distant metastasis (36); however, our study did not identify them as independent predictors of distant metastasis. This discrepancy may be attributed to an inadequate sample size.

To enhance the richness of image information acquisition, this study employs a Laplacian of Gaussian (LoG) filter and a wavelet filter in the feature extraction process. The LoG filter, a second-order derivative filter, is primarily utilized for edge detection and feature point recognition (37). Conversely, the wavelet filter offers multi-scale and multi-directional analytical capabilities, and is extensively applied in denoising and image enhancement techniques (38). It is evident that the majority of the 15 selected features are derived from these two filters. Moreover, the Spearman correlation coefficient is applied to eliminate features with high inter-correlation, which aids in reducing the complexity of the model and mitigating the risk of overfitting. Subsequently, the radiomics models are established using machine learning algorithms such as SVM, LR, RF, NB, and XGB, based on the selected features. The findings suggest that LR, SVM, and RF-based radiomics models demonstrate comparable predictive efficacy as clinical features, thereby establishing themselves as dependable biomarkers for forecasting GC metastasis.

To the best of our knowledge, this study represents the first attempt to develop and validate a radiomics-based nomogram for predicting the risk of distant metastases in patients with GC. The utility of this approach lies in its utilization of readily available CT imaging technology and a well-established methodology to forecast the risk of distant metastases, without incurring additional financial burden for patients. Furthermore, the presentation of the model in a nomogram format allows for a more graphic representation of how various parameters influence the outcomes, thus facilitating its clinical application. However, several limitations of our research should be acknowledged. Firstly, despite the robust performance of the model, it requires a necessary suite of external validations to corroborate its generalizability. Consequently, future studies should incorporate a diverse patient population from different centers for validation purposes. Secondly, the current study is retrospective in nature and hence necessitates confirmation through additional prospective investigations. Lastly, given our selection of venous phase CT images for ROI segmentation, the predictive capabilities of extracting features from arterial phase and delayed phase CT images require additional verification.

## 5 Conclusions

Radiomics of venous phase CT images prior to treatment holds promise as a potential biomarker for predicting the occurrence of distant metastases in individuals diagnosed with gastric cancer. The radiomics-clinical model exhibits a remarkable predictive capability, offering significant value in the early identification of high-risk patients with a propensity for distant metastases, thereby providing a broad application potential in clinical decision-making.

## Data Availability

The raw data supporting the conclusions of this article will be made available by the authors, without undue reservation. Requests to access these datasets should be directed to H-BX, 1344206467@qq.com.

## References

[1] Sung H Ferlay J Siegel RL Laversanne M Soerjomataram I Jemal A. Global cancer statistics 2020: GLOBOCAN estimates of incidence and mortality worldwide for 36 cancers in 185 countries. CA Cancer J Clin. (2021) 71:209–49. doi: 10.3322/caac.21660 33538338

[2] Ajani JA D'amico TA Bentrem DJ Chao J Cooke D Corvera C. Gastric cancer, version 2.2022, NCCN clinical practice guidelines in oncology. J Natl Compr Canc Netw. (2022) 20:167–92. doi: 10.6004/jnccn.2022.0008 35130500

[3] Liu J Sun R Cai K Xu Y Yuan W. A nomogram combining neutrophil to lymphocyte ratio (NLR) and prognostic nutritional index (PNI) to predict distant metastasis in gastric cancer. Sci Rep. (2024) 14:15391. doi: 10.1038/s41598-024-65307-7 38965325 PMC11224267

[4] Zhang X Wang X Li W Sun T Diao D Dang C. Predictive value of neutrophil-to-lymphocyte ratio for distant metastasis in gastric cancer patients. Sci Rep. (2022) 12:10269. doi: 10.1038/s41598-022-14379-4 35715490 PMC9205918

[5] Zhang Y Lin Y Duan J Xu K Mao M Wang X. A population-based analysis of distant metastasis in stage IV gastric cancer. Med Sci Monitor. (2020) 26:e923867. doi: 10.12659/MSM.923867 PMC7245058 32409630

[6] Kwee RM. Modern imaging techniques for preoperative detection of distant metastases in gastric cancer. World J Gastroenterol. (2015) 21(37):10502–9. doi: 10.3748/wjg.v21.i37.10502 PMC4588073 26457011

[7] Gillies RJ Kinahan PE Hricak H. Radiomics: images are more than pictures, they are data. Radiology. (2016) 278:563–77. doi: 10.1148/radiol.2015151169 PMC4734157 26579733

[8] Ligero M Garcia-Ruiz A Viaplana C Villacampa G Raciti MV Landa J. A CT-based radiomics signature is associated with response to immune checkpoint inhibitors in advanced solid tumors. Radiology. (2021) 299:109–19. doi: 10.1148/radiol.2021200928 33497314

[9] Aerts HJ Velazquez ER Leijenaar RT Parmar C Grossmann P Carvalho S. Decoding tumour phenotype by noninvasive imaging using a quantitative radiomics approach. Nat Commun. (2014) 5:4006. doi: 10.1038/ncomms5006 24892406 PMC4059926

[10] Aerts HJ. The potential of radiomic-based phenotyping in precision medicine: A review. JAMA Oncol. (2016) 2:1636–42. doi: 10.1001/jamaoncol.2016.2631 27541161

[11] Lambin P Leijenaar RTH Deist TM Peerlings J de Jong EEC van Timmeren J. Radiomics: the bridge between medical imaging and personalized medicine. Nat Rev Clin Oncol. (2017) 14:749–62. doi: 10.1038/nrclinonc.2017.141 28975929

[12] Gao X Ma T Cui J Zhang Y Wang L Li H. A CT-based radiomics model for prediction of lymph node metastasis in early stage gastric cancer. Acad Radiol. (2021) 28:e155–e64. doi: 10.1016/j.acra.2020.03.045 32507613

[13] Zhang QW Yang PP Gao YJ Li ZH Yuan Y Li SJ. Assessing synchronous ovarian metastasis in gastric cancer patients using a clinical-radiomics nomogram based on baseline abdominal contrast-enhanced CT: a two-center study. Cancer Imaging. (2023) 23:71. doi: 10.1186/s40644-023-00584-5 37488597 PMC10367237

[14] Dong D Tang L Li ZY Fang MJ Gao JB Shan XH. Development and validation of an individualized nomogram to identify occult peritoneal metastasis in patients with advanced gastric cancer. Ann Oncol. (2019) 30:431–8. doi: 10.1093/annonc/mdz001 PMC6442651 30689702

[15] Wu A Wu C Zeng Q Cao Y Shu X Luo L. Development and validation of a CT radiomics and clinical feature model to predict omental metastases for locally advanced gastric cancer. Sci Rep. (2023) 13(1):8442. doi: 10.1038/s41598-023-35155-y 37231100 PMC10213037

[16] Van Griethuysen JJM Fedorov A Parmar C Hosny A Aucoin N Narayan V. Computational radiomics system to decode the radiographic phenotype. Cancer Res. (2017) 77:e104–e7. doi: 10.1158/0008-5472.CAN-17-0339 PMC5672828 29092951

[17] Van Calster B Wynants L Verbeek JFM Verbakel JY Christodoulou E Vickers AJ. Reporting and interpreting decision curve analysis: A guide for investigators. Eur Urol. (2018) 74:796–804. doi: 10.1016/j.eururo.2018.08.038 30241973 PMC6261531

[18] Barbour SJ Coppo R Zhang H Liu ZH Suzuki Y Matsuzaki K. Evaluating a new international risk-prediction tool in igA nephropathy. JAMA Intern Med. (2019) 179:942–52. doi: 10.1001/jamainternmed.2019.0600 PMC6583088 30980653

[19] Zhou ZR Wang WW Li Y Jin KR Wang XY Wang ZW. In-depth mining of clinical data: the construction of clinical prediction model with R. Ann Transl Med. (2019) 7:796. doi: 10.21037/atm.2019.08.63 32042812 PMC6989986

[20] Leeman MF Patel D Anderson J OʼNeill JR Paterson-Brown S. Multidetector computed tomography versus staging laparoscopy for the detection of peritoneal metastases in esophagogastric junctional and gastric cancer. Surg Laparosc Endosc Percutan Tech. (2017) 27:369–74. doi: 10.1097/SLE.0000000000000451 28787380

[21] Feng XY Wang W Luo GY Wu J Zhou ZW Li W. Comparison of endoscopic ultrasonography and multislice spiral computed tomography for the preoperative staging of gastric cancer - results of a single institution study of 610 Chinese patients. PloS One. (2013) 8:e78846. doi: 10.1371/journal.pone.0078846 24223855 PMC3815220

[22] Pan Z Zhang H Yan C Du L Ding B Song Q. Determining gastric cancer resectability by dynamic MDCT. Eur Radiol. (2010) 20:613–20. doi: 10.1007/s00330-009-1576-2 19707768

[23] Warkentin MT Al-Sawaihey H Lam S Liu G Diergaarde B Yuan JM. Radiomics analysis to predict pulmonary nodule Malignancy using machine learning approaches. Thorax. (2024) 79:307–15. doi: 10.1136/thorax-2023-220226 PMC10947877 38195644

[24] Cicalini I Chiarelli AM Chiacchiaretta P Perpetuini D Rosa C Mastrodicasa D. Multi-omics staging of locally advanced rectal cancer predicts treatment response: a pilot study. Radiol Med. (2024) 129:712–26. doi: 10.1007/s11547-024-01811-0 PMC11088547 38538828

[25] Feng JW Liu SQ Qi GF Ye J Hong LZ Wu WX. Development and validation of clinical-radiomics nomogram for preoperative prediction of central lymph node metastasis in papillary thyroid carcinoma. Acad Radiol. (2024) 31:2292–305. doi: 10.1016/j.acra.2023.12.008 38233259

[26] Liu S Deng J Dong D Fang M Ye Z Hu Y. Deep learning-based radiomics model can predict extranodal soft tissue metastasis in gastric cancer. Med Phys. (2023) 51(1):267–277. doi: 10.1002/mp.16647 37573524

[27] Sun Z Zhang T Ahmad MU Zhou Z Qiu L Zhou K. Comprehensive assessment of immune context and immunotherapy response via noninvasive imaging in gastric cancer. J Clin Invest. (2024) 134(6):e175834. doi: 10.1172/JCI175834 38271117 PMC10940098

[28] Wang W Peng Y Feng X Zhao Y Seeruttun SR Zhang J. Development and validation of a computed tomography–based radiomics signature to predict response to neoadjuvant chemotherapy for locally advanced gastric cancer. JAMA Network Open. (2021) 4(8):e2121143. doi: 10.1001/jamanetworkopen.2021.21143 34410397 PMC8377567

[29] Jiang Y Wang H Wu J Chen C Yuan Q Huang W. Noninvasive imaging evaluation of tumor immune microenvironment to predict outcomes in gastric cancer. Ann Oncol. (2020) 31:760–8. doi: 10.1016/j.annonc.2020.03.295 32240794

[30] Ji T Zhou F Wang J Zi L. Risk factors for lymph node metastasis of early gastric cancers in patients younger than 40. Med (Baltimore). (2017) 96:e7874. doi: 10.1097/MD.0000000000007874 PMC5604635 28906366

[31] Takatsu Y Hiki N Nunobe S Ohashi M Honda M Yamaguchi T. Clinicopathological features of gastric cancer in young patients. Gastric Cancer. (2016) 19:472–8. doi: 10.1007/s10120-015-0484-1 25752270

[32] Qian S Golubnitschaja O Zhan X. Chronic inflammation: key player and biomarker-set to predict and prevent cancer development and progression based on individualized patient profiles. Epma J. (2019) 10:365–81. doi: 10.1007/s13167-019-00194-x PMC6882964 31832112

[33] Moses K Brandau S. Human neutrophils: Their role in cancer and relation to myeloid-derived suppressor cells. Semin Immunol. (2016) 28:187–96. doi: 10.1016/j.smim.2016.03.018 27067179

[34] Coupland LA Parish CR. Platelets, selectins, and the control of tumor metastasis. Semin Oncol. (2014) 41:422–34. doi: 10.1053/j.seminoncol.2014.04.003 25023359

[35] Ray-Coquard I Cropet C Van Glabbeke M Sebban C Le Cesne A Judson I. Lymphopenia as a prognostic factor for overall survival in advanced carcinomas, sarcomas, and lymphomas. Cancer Res. (2009) 69:5383–91. doi: 10.1158/0008-5472.CAN-08-3845 PMC2775079 19549917

[36] Abu-Shawer O Abu-Shawer M Haimour A Alhouri A Alkhatib AA Abki M. Hematologic markers of distant metastases in gastric cancer. J Gastrointest Oncol. (2019) 10:529–36. doi: 10.21037/jgo.2019.01.12 PMC6534726 31183204

[37] Nonato LG Do Carmo FP Silva CT. GLoG: laplacian of gaussian for spatial pattern detection in spatio-temporal data. IEEE Trans Vis Comput Graph. (2021) 27:3481–92. doi: 10.1109/TVCG.2020.2978847 32149640

[38] Penedo SRM Netto ML Justo JF. Designing digital filter banks using wavelets. EURASIP J Adv Signal Process. (2019) 2019(1):1–11. doi: 10.1186/s13634-019-0632-6

