# Supplementary material for: Development and validation of an individualized nomogram for predicting distant metastases in gastric cancer using a CT radiomics-clinical model
Source: Front Oncol. 2024 Nov 29;14:1476340. doi: 10.3389/fonc.2024.1476340 (PMC11672336; doi:10.3389/fonc.2024.1476340)
Supplement: Supplementary file 1 [file DataSheet1.docx]

Supplementary Material

# Supplementary Data

Data 1. The calculation formula of radscore.

**Radscore** = 1.0511196021331402 ＋

(0.000362671387223514 × original_shape_Maximum2DDiameterRow) -

(0.8552818225456186 × original_glcm_Imc2) ＋

(0.11874866803562467 × log-sigma-3.0-mm-3D_glcm_MCC) ＋

(0.00039338815659244934 × log-sigma-3.0-mm-3D_glszm_HighGrayLevelZoneEmphasis) -

(23.023822786472397 × log-sigma-3.0-mm-3D_glszm_SmallAreaLowGrayLevelEmphasis) ＋

(16.105143418436572 × wavelet-LLH_firstorder_Median) ＋

(5.3505087328870775e-05 × wavelet-LLH_glrlm_LongRunHighGrayLevelEmphasis) -

(0.002066359144272759 × wavelet-LLH_glszm_GrayLevelVariance) ＋

(0.0001931193114041553 × wavelet-LLH_glszm_HighGrayLevelZoneEmphasis) -

(0.24156188978353985 × wavelet-LLH_glszm_SmallAreaEmphasis) -

(0.0009212963978901822 × wavelet-HLH_glszm_SmallAreaHighGrayLevelEmphasis) ＋

(9.644924614966758 × wavelet-HHL_glcm_Imc1) ＋

(2.1704352740597818e-08 × wavelet-LLL_firstorder_TotalEnergy) ＋

(1.3566338123242139 × wavelet-LLL_firstorder_Variance) ＋

(0.31595978859833906 × wavelet-LLL_glcm_InverseVariance)

# Supplementary Figures and Tables

## Supplementary Table

Table 1. The detailed information for all features.

| **Features** | | **Numbers** |
| --- | --- | --- |
| **Firstorder** | | **18** |
| **Shape** | | **14** |
| **Texture** | **Glcm (GrayLevelCooccurenceMatrix)** | **24** |
|  | **Glrlm (GrayLevelRunLengthMatrix)** | **16** |
|  | **Glszm (GrayLevelSizeZoneMatrix)** | **16** |
|  | **Gldm (GrayLevelDependenceMatrix)** | **14** |
|  | **Ngtdm (NeighbouringGrayToneDifferenceMatrix)** | **5** |
| **LoG-sigma3.0mm** | | **93** |
| **Wavelet** | **Wavelet-LLL** | **93** |
|  | **Wavelet-HLL** | **93** |
|  | **Wavelet-LHL** | **93** |
|  | **Wavelet-HHL** | **93** |
|  | **Wavelet-LLH** | **93** |
|  | **Wavelet-HLH** | **93** |
|  | **Wavelet-LHH** | **93** |
|  | **Wavelet-HHH** | **93** |

Table 2. Configuration Settings during feature extraction.

| **Parameter** | **Settings** |
| --- | --- |
| Python Versions | 3.11.4 |
| Minimum ROI Dimensions | 2 |
| Minimum ROI Size | None |
| Normalize | True |
| normalizeScale | 1 |
| removeOutliers | None |
| resampledPixelSpacing | None |
| interpolator | 1 |
| preCrop | False |
| padDistance | 5 |
| distances | [1] |
| force2D | False |
| force2Ddimension | 0 |
| resegmentRange | None |
| additionalInfo | True |
| binCount | 32 |
| resamplePixelSpacing | [2, 2, 2] |
